# Women’s utilisation of prevention of mother-to-child transmission of human immunodeficiency virus services in Addis Ababa, Ethiopia

**DOI:** 10.4102/hsag.v23i0.1145

**Published:** 2018-08-27

**Authors:** Tefera G. Negash, Valerie J. Ehlers

**Affiliations:** 1 Department of Health Studies, University of South Africa, South Africa

## Abstract

**Background:**

Human immunodeficiency virus (HIV) mother-to-child transmission (MTCT) can be prevented when HIV-positive pregnant women use effective prevention of mother-to-child transmission (PMTCT) of HIV services. Approximately 50% of HIV-positive pregnant women used free PMTCT services in Ethiopia.

**Aim:**

This study attempted to identify factors influencing women’s utilisation of PMTCT services. Addressing such factors could enable more Ethiopian women to use PMTCT services. The study investigated whether women’s utilisation of services was affected by socio-demographic issues, their partners’ known HIV status, disclosure of their HIV-positive status, stigma and discrimination, and satisfaction with services.

**Setting:**

Prenatal clinics in Addis Ababa, Ethiopia.

**Methods:**

A quantitative, cross-sectional study design was used and 384 questionnaires were completed by women who used PMTCT services in Addis Ababa.

**Results:**

No socio-demographic characteristic prevented women’s utilisation of PMTCT services, nor did stigma, discrimination or disclosure of their HIV-positive status. Most respondents’ partners with unknown HIV status did not know that the respondents used PMTCT services. Most women were satisfied with the PMTCT services.

**Conclusions:**

Prevention of mother-to-child transmission services should remain accessible to all HIV-positive women in Ethiopia. Concurrent HIV partner testing should be encouraged with appropriate counselling. HIV-positive pregnant women should be encouraged to disclose their status to their partners so that they need not use PMTCT services secretly. Patients’ high levels of satisfaction with PMTCT services are a good indicator for rolling out PMTCT initiatives at other facilities. Future research should focus on HIV-positive pregnant women who do not use PMTCT services.

## Introduction

Human immunodeficiency virus can be transmitted from an infected mother to her baby during pregnancy, childbirth and breastfeeding. In the absence of interventions for prevention of mother-to-child transmission (PMTCT) of HIV, the probability of mother-to-child transmission of HIV (MTCT) could be 35% in developing countries, but effective PMTCT interventions could reduce MTCT to less than 5% among breastfed babies (World Health Organization [WHO] 2010:12). Survival rates and the quality of life of HIV-positive persons can be enhanced through sustained chronic care and treatment for HIV-infected pregnant or postpartum women and their HIV-exposed and HIV-infected children (WHO 2011a:1–12). Prevention of mother-to-child transmission services refer to the reduction of new paediatric HIV infections by promoting primary prevention of HIV among women and men of reproductive age, addressing family planning within the context of HIV, promoting access to HIV and antiretroviral treatment (ART), providing prophylaxis for HIV-infected pregnant women and their families, and promoting access of HIV-exposed infants to care (Ethiopian Federal Ministry of Health [EFMOH] 2011:3). In this study, ‘PMTCT services’ refers to the utilisation of prophylactic antiretrovirals (ARVs) or ART by HIV-positive pregnant women, depending on their CD4 counts. Appropriate infant feeding practices comprise an integral part of PMTCT services but are not addressed in this article.

During 2011, 34 million people were living with HIV globally. Sub-Saharan Africa (SSA) was the most severely affected region with 4.9% HIV prevalence, accounting for 69.0% of all people living with HIV worldwide. Worldwide in 2011, 1.7 million people died from AIDS-related causes (Joint United Nations Program on HIV/AIDS [UNAIDS] 2012:6–12). The global number of people living with HIV or AIDS in 2012 was an estimated 35.3 million as a result of ARVs. Globally, the use of ARVs for PMTCT increased from 57% in 2011 to 63% in 2012. Globally, PMTCT services prevented an estimated 670 000 children from being infected with HIV from 2009 to 2012 (UNAIDS 2013:4, 38–40).

The WHO’s global plan hoped to attain ‘virtual’ (implying ‘almost complete’) elimination of new HIV infections among children and improve the health of mothers by 2015. This global plan aims to reduce new HIV infections among children by 90%; decrease the number of women dying from HIV-associated conditions during pregnancy, childbirth and postpartum by 50%; and lower MTCT to less than 5%. During 2010, 35% of pregnant women in low- and middle-income countries received HIV testing and counselling (HTC) compared to 26% in 2009. In SSA this coverage increased from 35% to 42%, with the highest increase in Eastern and Southern Africa (from 52% to 61%). During 2010, the coverage of effective regimens for PMTCT was 48%. Out of the estimated 1.49 million infants born to HIV-infected mothers, 42% received ARVs to prevent HIV infections (WHO 2011b:139).

The PMTCT services in Ethiopia underachieved according to the EFMOH. In Ethiopia, PMTCT services remain underutilised, with a reported effective coverage of only 1.1% in some areas (Hussein, Jira & Girma 2011:6). According to the EFMOH’s progress report, the number of health facilities providing PMTCT services had increased from 32 in 2004 to 1352 in 2010 and to 1445 by the end of June 2011. However, in 2011, the percentage of HIV-positive pregnant women who used ARVs was only 24%, indicating missed opportunities to reduce the impact of MTCT and HIV or AIDS in Ethiopia (EFMOH 2012:30).

Ethiopia’s PMTCT services reportedly reached less than 50% coverage of HIV-positive pregnant women in 2012 (UNAIDS 2013:4, 38–40). During the same year, the reported HIV prevalence in Ethiopia was 1.5% among adults aged 15–49. Among men the prevalence was 1.0% while among women it was 1.9%. However, the prevalence in Addis Ababa was higher, at 5.2%, with the prevalence among men being 4.3% and among women 6.0% (UNAIDS 2013:4, 38–40). The HIV prevalence rate among women attending antenatal care (ANC) clinics during the preceding 3 years in the public sector was 1.7%, while it was 3.1% outside the public sector (Central Statistical Agency of Ethiopia & Inner City Fund International [CSAE/ICFI] 2012:232–236). Because Addis Ababa has a relatively high HIV prevalence compared to Ethiopia’s national HIV prevalence, research in Addis Ababa is essential to identify strengths and weaknesses of HIV prevention and treatment services and specifically to identify factors influencing pregnant women’s utilisation of PMTCT services, impacting on the number of HIV-negative babies born to HIV-positive mothers.

### Statement of the research problem

Free PMTCT services are available in Addis Ababa, which could reduce the risk of MTCT considerably. Many HIV-positive pregnant women do not use the available free PMTCT services, increasing the risk of MTCT for their babies and depriving themselves of opportunities to enhance their own health status. As HIV-positive pregnant women who did not use PMTCT services comprised an inaccessible population, this study’s research problem was to identify factors that might have influenced HIV-positive pregnant women to use PMTCT services in Addis Ababa.

### Purpose and objectives

The purpose of this study was to identify whether socio-demographic, personal or service-related factors affected the utilisation of PMTCT services in Addis Ababa, as less than 50% of HIV-positive pregnant women used these services during 2012 (UNAIDS 2013:4). If strengths and weaknesses of the PMTCT programme could be identified, then recommendations could be provided for enhancing the utilisation of these services.

The objectives of this study were to identify whether the following aspects, identified during an in-depth literature review, influenced HIV-positive women’s utilisation of PMTCT services in Addis Ababa:

- socio-demographic characteristics;
- partners’ known HIV status;
- disclosure of the respondents’ HIV-positive status;
- stigma and discrimination;
- satisfaction with the quality of PMTCT services in Addis Ababa.

### Definitions of key concepts

*Acquired immune deficiency syndrome (AIDS)* refers to HIV clinical stage 3 or 4 disease or, where CD4 is available, any clinical stage and CD4 < 350 cells/mm^3^ (WHO 2005:10).

*Antenatal care (ANC)* implies a service provided to pregnant women including at least four focused ANC clinic visits, with the first visit as early as possible during pregnancy, the second visit at 28–32 weeks, the third visit after 36 weeks and the fourth one before the expected date of delivery. During the first ANC visit routine provider-initiated HTC should be provided in order to identify HIV-positive pregnant women and to offer PMTCT services (EFMOH 2011:11).

*Antiretrovirals (ARVs)* are medications that cannot cure HIV but they restore immune function, suppress viral replication and reduce HIV-related morbidity and mortality. Therefore, ARVs can prolong HIV-positive persons’ lives and enhance the quality of their lives (EFMOH 2008:48). In this study, ARVs refer to temporary prophylactic use of ARVs while pregnant and breastfeeding to reduce the risk of MTCT, prescribed if the woman’s CD4 count is equal to or greater than 350 cells/mm^3^. However, if the woman’s CD4 count is below 350 cells/mm^3^, then lifelong ART should be prescribed.

*HIV testing and counselling (HTC)* refers to counselling, testing and announcing of HIV test results to a client. If the client tests HIV-positive, it can be linked to appropriate care and treatment services. It has two components: voluntary testing and counselling and provider-initiated testing and counselling (WHO 2009:7).

*Human immunodeficiency virus (HIV)* is a virus that damages the body’s immune system, the system that fights infections. Human immunodeficiency virus is an aetiologic agent of AIDS and can be transmitted by sexual contact, from blood and blood products such as needle stick injuries, sharing injection needles or instruments used in tattooing or circumcision and by HIV-infected mothers to their infants during pregnancy, birth or breastfeeding (Fauci & Lane 2005:1079).

*Mother-to-child transmission of HIV* can occur from an HIV-positive mother to her baby during pregnancy, childbirth and breastfeeding.

*Prevention of mother-to-child transmission (PMTCT)* was defined and explained in the first paragraph of the introduction.

## Method

A quantitative, cross-sectional design was used. In Addis Ababa, PMTCT services are provided by 61 health care facilities (10 public hospitals, 15 private hospitals and 36 public health centres), comprising the site population for this study (EFMOH 2012:1). The names of the 61 health facilities were written on slips of paper and placed into three boxes representing the three types of health care facilities. Proportional stratified random sampling of the sites was done by blindly drawing two names from the public hospitals, three from the private hospitals and seven from the public health centres.

Women who used PMTCT services in Addis Ababa (*N* = 796) from May till November 2013 comprised the study population. The sample size of 384 was calculated using a single proportion formula:

*S* = *p*(1–*p*)*z*^2^/*d*^2^

where *p* stands for anticipated population proportion, *z* refers to the cut-off value of the normal distribution and *d* is the precision required on either side of the proportion. The total number of women who used PMTCT services at each of the 12 participating facilities was used to determine the number of respondents at each clinic, amounting to proportional stratified sampling. At each site, the PMTCT clients’ medical record numbers were used as census. Then a table of random numbers was used to randomly select 20% of the women at each site. The selected women were contacted during their follow-up visits to the PMTCT service sites, informed about the study and requested to participate by answering questions read to them from a questionnaire.

### Reliability and validity of the research instrument

The Cronbach alpha coefficient exceeded 0.70 for all items, indicating an acceptable internal reliability (Burns & Grove 2005:399). A *p*-value of 0.05 was used as a cut-off value for any significant association at a confidence interval (CI) of 95%.

Content validity can be expressed numerically using a content validity index.

Five experts, working in PMTCT services in Addis Ababa, rated the content relevance of each item using a four-point rating scale (1 = not relevant; 2 = unable to assess relevance without item revision or item requiring such revision that it would no longer be relevant; 3 = relevant but needs minor alteration; 4 = very relevant and succinct). These experts were also requested to identify aspects not included in the instrument (Burns & Grove 2005:378). Only items that scored three or higher were included in the instrument. For face validity these experts had to agree that the questionnaire’s items requested information about PMTCT use. An appropriately defined population, a large sample size and a proportional stratified random sampling technique enhanced the external validity of the study, implying that the findings for this study could be generalised to the population of women using PMTCT services in Addis Ababa.

### Data collection

The instrument was pretested by 19 women (who were excluded from the actual study), who replied to the questionnaire’s items. Based on the results of the pretest, questions pertaining to stigma and discrimination were rephrased to use more familiar Amharic words. The actual data collection took place from May to November 2013. Three research assistants, fluent in both English and Amharic, were trained for 2 days to ask the questions in face-to-face situations and to record the respondents’ verbatim responses in Amharic. The research assistants signed confidentiality agreements with the principal investigator. The first author translated the questionnaire into Amharic and the respondents’ verbatim Amharic statements into English. Thereafter a professional Amharic–English translator confirmed that the translations were accurate.

### Ethical considerations

Ethical considerations involved obtaining permission from the relevant health and academic authorities to conduct the study and ensure that the respondents were treated with respect.

The head of each participating health facility granted permission to collect data from PMTCT clients and every respondent signed an informed consent form. Confidentiality was maintained as only the researchers and statistician had access to the completed questionnaires. Data collection took place in private rooms at the clinics. The first author was available throughout the data collection procedures and could attend to any woman who might require assistance during or after the session with a research assistant, but such assistance was never required. No names were used on the questionnaires. The first author kept a list linking every respondent’s number to her medical file number to avoid contacting any women more than once. This list was only available to one person and would be destroyed with the completed questionnaires subsequent to the acceptance of the research report. Information transcribed from the completed questionnaires to a computer program was protected by a unique password on a computer to which only the researchers and the statistician had access.

### Data analysis

Descriptive and inferential statistics were used in the analysis of the data including the mean (average value) and chi-squared test (*χ*^2^). The *χ*^2^ analyses nominal data by comparing the expected frequencies with the observed frequencies of data sets (Burns & Grove 2005:791). Cramer’s V is used to analyse nominal data where the contingency tables are larger than 2 × 2 (Burns & Grove 2005:794). The *p-*value indicates the probability that a specific event will occur and is calculated by dividing the total number of events (*N*) by the number of events with the relevant outcomes (*n*). As such a *p*-value can range from 0 (no probability) to 1 (perfect probability or 100% certainty) that a specific outcome will occur, as explained by Burns and Grove (2005:137–138). A *p*-value of 0.05 and a CI (‘… a range in which the parameter of value is estimated to be’ [Burns & Grove 2005:793]) of 95% were used as cut-off values for associations. The Statistical Package for the Social Sciences (SPSS) version 20, EpiInfo version 3.51 and Microsoft Excel 2007 programs were used to conduct the data analysis. A statistician assisted with the data analysis and interpretation and also rendered inputs during the development of the research instrument.

### Ethical considerations

Ethical clearance was granted by the University of South Africa (certificate number HSHDC/122/2012) and by the Addis Ababa City Administration Health Bureau Ethical Clearance Committee.

## Results

### Socio-demographic characteristics

The average age of the women who used PMTCT services was 28.1 years, ranging from 19 to 38. The mode was 26–30 years (46.9%; *f* = 180). Most respondents were married (88.5%; *f* = 340). As many as 37.7% (*f* = 145) of the respondents attained Grades 9–12, followed by Grades 7–8 (24.7%; *f* = 95). Exactly 5.7% (*f* = 22) of the women never attended school and 14.6% (*f* = 56) attained university or college level education.

Most respondents (94.6%; *f* = 53) with university or college level education knew their HIV status before their initial presentation at ANC clinics while 77.9% (*f* = 74) of those who attained Grades 7–8 knew their HIV status at that stage. This difference was statistically significant (*p* = 0.05; *χ*^2^ = 0.035) Cramer’s V showed a weak association (*V* = 0.164). Most married women (86.5%; *f* = 294) knew their HIV-positive status before commencing with their ANC visits, while fewer pregnant women who had never been married (80.0%; *f* = 8), were separated (66.7%; *f* = 14) or divorced (0.0%; *f* = 0) did so. This difference was statistically significant (*p* = 0.05; χ^2^ = 0.003). Among self-employed women, 91.8% (*f* = 67) knew their HIV status before presentation at the ANC clinic but only 60.7% (*f* = 17) of the part-time employed respondents had this knowledge, which was statistically significant (*p* = 0.05; χ^2^ = 0.001).

**FIGURE 1:**
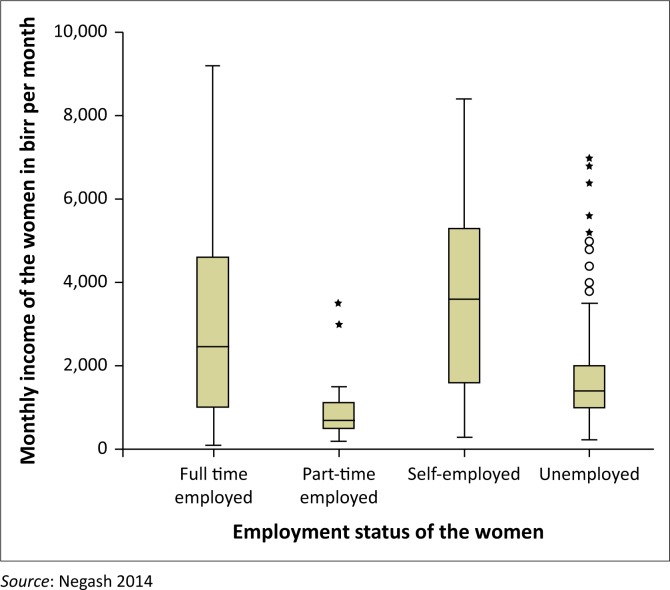
Employment status versus monthly birr incomes of respondents (*N* = 384).

As indicated in Figure 1, 39.6% (*f* = 152) of the respondents were employed full-time, 7.3% (*f* = 28) were employed part-time, 19.0% (*f* = 73) were self-employed and 34.1% (*f* = 131) were unemployed. The average monthly income of full-time employed women was 2909 birr per month (95% CI = 1632.3–3313.654) and the average monthly income of self-employed women was 3747 birr per month (95% CI = 3207–4287). Monthly incomes were the highest for those women who were self-employed and the second highest for those who were employed full-time, while the part-time employed women earned the lowest monthly incomes. This difference was statistically significant using ANOVA (*p* < 0.01). All respondents had monthly incomes, possibly in the form of support from others in the case of the unemployed women. This might explain why respondents with different employment status managed to use PMTCT services.

Table 1 indicates that χ^2^ findings were significant for respondents’ education levels, marital status and employment status versus their knowledge about their HIV status when they commenced attending ANC clinics. However, Cramer’s V showed only weak associations in all three instances.

**TABLE 1:** Chi-square tests of respondents’ education level, marital status and employment status versus knowledge of Human immunodeficiency virus status when commencing antenatal care clinic visits (*N* = 384).

Variable	Value	df	p	Cramer’s V
Educational level versus knowledge of HIV status	10.321	4	0.035	0.164
Marital status versus knowledge of HIV status	17.909	5	0.003	0.216
Employment status versus knowledge of HIV status	17.306	3	0.001	0.212
Number of valid cases	384	-	-	-

*Source*: Negash 2014

HIV, Human immunodeficiency virus; df, degrees of freedom; *p*, probability.

### Human immunodeficiency virus status of respondents’ male partners

Husbands and male partners were not interviewed but the respondents were questioned about their partners’ HIV status. Out of the respondents’ male partners, 81.5% (*f* = 313) were HIV-positive, 7.3% (*f* = 28) were HIV-negative and 11.2% (*f* = 43) did not know their HIV status. Reportedly 88.5% (*f* = 340) of the HIV-positive pregnant women’s partners knew they were using PMTCT services but 11.5% (*f* = 44) did not know. Most HIV-positive male partners (97.8%; *f* = 306) knew that their female partners were using PMTCT services and only 2.2% (*f* = 7) did not know. Most HIV-negative male partners (92.9%; *f* = 26) also knew that these women were using PMTCT service, as only 7.1% (*f* = 2) were unaware of this situation. However, of the male partners with unknown HIV status, only 18.6% (*f* = 8) were aware and 81.4% (*f* = 35) were unaware that the respondents used PMTCT services. Thus, the highest percentage of HIV-positive male partners were aware that their partners used PMTCT services and the lowest percentage of male partners with unknown HIV status were aware of their partners’ utilisation of PMTCT services. This finding was statistically significant (χ^2^ < 0.01). Cramer’s V also showed a strong association between male partners’ known HIV status and their awareness of the respondents’ utilisation of PMTCT services (*V* = 0.781).

### Disclosure of respondents’ human immunodeficiency virus-positive status

Potential factors that could influence HIV-positive women’s utilisation of PMTCT services included marital status versus disclosure of HIV status, stigma and discrimination versus most recent CD4 count and employment status versus monthly income.

Most respondents (88.5%; *f* = 340) were married and had disclosed their HIV status, which is statistically significant (χ^2^ = 0.002). However, Cramer’s V showed a weak association (*V* = 0.225). Thus respondents’ disclosure of their HIV-positive status did not influence their utilisation of PMTCT services.

### Stigma and discrimination

Perceived stigma was the highest among those respondents who indicated that people acted as if being HIV-positive was the person’s own fault (18.8%; *f* = 72). Most respondents (92.2%; *f* = 354) disagreed that some people physically moved away from them once they learned that the women were HIV-positive. Internalised stigma (feelings of being branded by others) was experienced by 66.7% (*f* = 256) of the respondents, who felt worthless. Some HIV-positive pregnant women (36.7%; *f* = 141) isolated themselves from friends or family members because they were HIV-positive. No respondents had been denied health services during the 12 months preceding data collection because of their HIV-positive status. Discrimination was reported by those respondents (5.7%; *f* = 22) who experienced verbal abuse. The number of women who faced perceived stigma and discrimination was lower than those women who had internalised stigma. This level of stigma and discrimination did not prevent the respondents from using PMTCT services.

### Respondents’ satisfaction with prevention of mother-to-child transmission services in Addis Ababa

Most respondents were satisfied with the health facilities’ cleanliness (92.5%; *f* = 355) and with the waiting rooms’ comfort (87.0%; *f* = 343), but 39.3% (*f* = 151) were dissatisfied with the wheelchair accessibility of these facilities. Thus most respondents’ were satisfied with the PMTCT services they utilised.

## Discussion

### Biographic information

A study conducted in north-western Ethiopia reported that out of 418 pregnant women, 46.7% were unable to read and write, 9.8% had passed Grades 1–6, 7.2% had attained Grades 7–8, 19.1% had attained Grades 9–12 and 11.5% had acquired education beyond Grade 12 (Moges & Amberbir 2011:109). Similar findings were reported by a study conducted in four SSA countries, Burkina Faso, Kenya, Malawi and Uganda, indicating that 18% of the respondents did not attend formal education and women at the highest educational level were more likely to be tested for HIV (Obermeyer et al. 2013:1113–1116). This was similar to the current study’s findings that many HIV-positive pregnant women had limited or no schooling but that some of these women had acquired post school qualifications. Better-educated women seemed to be more likely to know their HIV status before commencing their ANC clinic visits and to understand the advantages of using PMTCT services.

A study conducted in the Dilchora Hospital in eastern Ethiopia involving 234 ANC attendees reported that out of the respondents who had undergone HIV testing and who were potential PMTCT users, 98.3% were married while 1.7% were not married (Demissie, Deribew & Abera 2009:143). In this study, most HIV-positive women (88.5%; *f* = 340) who used PMTCT services in Addis Ababa were married. Thus being married did not seem to influence HIV-positive pregnant women’s utilisation of PMTCT services.

A study that assessed PMTCT uptake in Kenya correlated with the women’s employment status. Out of 2700 respondents, 70.3% were unemployed, of whom 20 knew they were HIV-positive prior to their pregnancies but 92.1% only received their HIV test results during their pregnancies. No significant difference was observed in employment status between HIV tested and untested women (Kinuthia et al. 2011:3). A study conducted in Arba Minch, Ethiopia found that most women were not in formal or self-employment. The average reported household income was 727 birr, with the highest being 7168 birr (Adedimeji et al. 2012:3–4). In this study, most HIV-positive pregnant women were employed and knew their HIV status prior to commencing their ANC clinic visits. Employment status did not influence HIV-positive women’s utilisation of PMTCT services in Addis Ababa in this study nor in previously reported studies.

### Human immunodeficiency virus status of respondents’ partners

An Indian study reported that women whose partners’ HIV status was negative or unknown were 2.69 times more likely to discontinue using PMTCT services than women whose partners’ HIV-positive status was known (Panditrao et al. 2011:595). An Ethiopian study reported that only 6% of the HIV-positive female respondents had disclosed their HIV status to their sex partners. Women with regular sex partners were more likely to disclose their status than women who did not have regular sex partners (Kassaye, Lingerh & Dejene 2005:128). In this study, most HIV-positive pregnant women knew their partners’ HIV status and most partners encouraged their female partners to use PMTCT services. However, those partners with unknown HIV status did not encourage the respondents to utilise PMTCT services.

### Stigma and discrimination

A systematic review from 12 SSA countries revealed that stigma influenced HIV-positive pregnant women not to use PMTCT services, based on the findings of both qualitative and quantitative studies (Gourlay et al. 2013:4–6). In this study only a few HIV-positive pregnant women reportedly encountered stigma and discrimination. However, as many as 66.7% (*f* = 256) of the respondents experienced internal stigma, but this did not prevent them from using PMTCT services.

### Respondents’ satisfaction with prevention of mother-to-child transmission services in Addis Ababa

A study conducted at six hospitals and six health centres in Ethiopia reported similar findings to those of this study, indicating that most patients were satisfied with the PMTCT services. That study’s satisfaction levels ranged from 76.50% to 90.57% (Bekele et al. 2008:44), while this study’s satisfaction levels exceeded 80.0%. The only aspect with which some of this study’s respondents were dissatisfied was the wheelchair accessibility of these facilities.

## Conclusions

More married, educated and self-employed women knew their HIV status prior to commencing ANC clinic visits than their counterparts, enabling them to decide to use PMTCT services early during their pregnancies. However, no biographic factor was identified that negatively influenced HIV-positive women’s utilisation of PMTCT services in Addis Ababa.

The respondents’ partners whose HIV status was known were more likely to know about their utilisation of PMTCT services. Disclosure of HIV-positive status did not influence most women’s decisions to use PMTCT services as 88.5% (*f* = 340) had disclosed their status.

A minority of respondents reportedly encountered stigma and discrimination. However, no respondents failed to access health care services, based on their HIV-positive status, during the 12 months preceding the data collection. Although 66.7% (*f* = 256) of this study’s respondents experienced internal stigma, this did not prevent them from using PMTCT services. Most respondents were satisfied with the PMTCT services.

The strengths of the EFMOH’s PMTCT programme included that this study’s respondents were satisfied with these services, accessed and utilised these services, and only a few experienced external stigma. The weaknesses of the EFMOH’s PMTCT programme, according to this study’s findings, included that service sites were not wheelchair accessible and that HIV testing of sex partners did not always occur. As 66.7% of the respondents experienced internalised stigma, this aspect might not have been adequately addressed by the PMTCT service providers.

## Recommendations

The findings could help to improve the PMTCT programme, develop guidelines, design policy and conduct further research. Therefore, PMTCT programme managers, health care providers, health science students, researchers and HIV/AIDS patients could benefit from this study’s findings.

Strengthening the educational curriculum to teach girls and women about PMTCT services is essential for women to make informed choices about the utilisation of these services. However, men and boys should also be informed about the benefits of PMTCT services so that they can support their female partners to use these services effectively.

It is essential to provide HIV prevention services both for the partner and the pregnant woman. Health provider initiated counselling and testing (HCT) sites should encourage all HIV-positive women to bring their partners for HIV testing and for couples’ counselling.

Health care providers should counsel every patient about HIV/AIDS, PMTCT, stigma and discrimination. They should identify HIV-positive women who develop internal stigma and refer them for counselling. Women who use PMTCT services should be supported to disclose their HIV status.

Future research should focus on women who do not use PMTCT services and those who discontinue using these services because they might have different experiences from HIV-positive women who use these services. Similar research should be conducted in other regions of Ethiopia in order to increase the national representativeness of the findings. More research is recommended by adding qualitative methods such as in-depth interviews and focus group discussions to gather information about the HIV-positive women’s lived experiences related to the utilisation of PMTCT services or factors influencing their decisions not to use these services.

Better-educated women were more empowered than their counterparts with no or primary school education. Consequently, enhanced education for girls and women is essential for empowering these women to use PMTCT services for their own and for their babies’ benefits.

In future, ANC clinics should be requested to identify HIV-positive pregnant women who do not use PMTCT services. Researchers should then investigate these women’s reasons for not utilising the free PMTCT services in Addis Ababa. If some of these identified barriers could be addressed, then more HIV-positive pregnant women in Addis Ababa might be enabled to use PMTCT services to their own and to their infants’ advantage.

## Limitations of the study

A major limitation of this study was the inability to study factors influencing HIV-positive pregnant women’s decisions not to utilise PMTCT services in Addis Ababa, as this was an inaccessible population. The experiences of this population of HIV-positive pregnant women might have differed from those of women who managed to use PMTCT services and who participated in this study.

Only questionnaires were used to collect information. Richer information might have been obtained if individual in-depth and focus group interviews had been conducted.

The respondents’ answers were accepted without implementing any checks to determine whether or not their answers were true. Such checks would have necessitated checking the respondents’ medical records and those of their babies and partners, which might have jeopardised the researchers’ promises of anonymity and confidentiality.
